# Body image perception and social support as predictors of psychological distress among third trimester pregnant women in Nigeria

**DOI:** 10.1186/s12884-024-06463-6

**Published:** 2024-04-22

**Authors:** Oluwaseyi Isaiah Olabisi, Eunice Ogunmodede, Simeon Ojo, Oluwafemi Ilori, Deborah T. Esan

**Affiliations:** 1 Department of Mental Health and Psychiatric Nursing, Faculty of Nursing Science, Bowen University, Iwo, Nigeria; 2 Department of Maternal and Child Health Nursing, Faculty of Nursing Science, Bowen University, Iwo, Nigeria; 3 Department of Medico-Surgical Nursing, Faculty of Nursing Science, Bowen University, Iwo, Nigeria; 4 Department of Community Health/Public Health Nursing, Faculty of Nursing Science, Bowen University, Iwo, Nigeria

**Keywords:** Body image perception, Pregnancy, Psychological distress, Social support, Third trimester

## Abstract

**Background:**

Body image perception and social support during pregnancy can impact the psychological distress levels experienced by pregnant women. As a result, the purpose of this study was to examine the relationship between various components of social support and body image perception on psychological distress levels among pregnant women in their third trimester in Nigeria.

**Method:**

A cross-sectional study was conducted among 246 pregnant women who were in the third trimester and attending selected health care facilities in Ogbomoso, a semiurban city in Oyo State, Nigeria. Body image perception, social support, and psychological distress scales were used to collect the data. Data were analyzed and summarized using descriptive and inferential statistics (ANOVA and multiple regression), with significance set at *p* < 0.05.

**Results:**

Regression analysis showed that 44% of the variation in psychological distress among pregnant women was explained by the background variables, marital status, body image perception, appraisal support, tangible support, belonging support, interaction between body image perception and appraisal support, belong support and tangible support.

**Conclusion:**

Intervention programs focusing on bolstering tangible support, belonging support and appraisal support are recommended at reducing the psychological distress due to body image perception among pregnant women at third trimester.

## Introduction

Women experience a variety of changes at all stages of pregnancy, and these affect every aspect of their lives, including the social, physical, and psychological domains. According to Watson et al. [1], pregnancy constitutes a time of significant life change requiring major psychological adjustment, which is often associated with anxiety and stress. Pregnant women experience psychological distress during pregnancy [2, 3], but it is more common during the third trimester, which may influence the risks of low birth weight, long-term cognitive defects, prolonged delivery, and preterm delivery [2, 4–6]. Fear of the unknown, potential birth trauma, concerns about neonatal developmental disorders, and concerns about changes in relationships with partners, family, and friends are some of the risk factors for a high level of psychological distress during the third trimester of pregnancy [3, 4, 7, 8].

A woman’s satisfaction with her body image is one of the factors that determines her physical and mental wellbeing, while a negative body image can lead to many health problems [9, 10]. It is quite obvious that pregnancy changes the body image of all women, which causes either positive or negative perceptions [11]. According to Bergmeier et al. [12], there are dramatic changes in a woman’s body shape that can be associated with body image concerns. Several alterations in outward appearance, namely, weight gain and skin changes, occur throughout the entire body during pregnancy. Sohrabi et al. [13] categorized the body image perception of pregnant women into three categories: symbols from motherhood and vulnerability; feelings towards changes in the body derived from negative feelings towards changes in the skin; unfit, ridiculous-obsessed shape and attention-seeking shape of the body; and attraction category from sexual with beauty attractions. Additionally, Goonapienuwala et al. [14] and Voelker et al. [15] averred that body image, as a multifaceted construct, includes perceptual, cognitive, and affective components regarding one’s own body as well as the bodies of others. Fahami et al. [16] and Plante et al. [17] concluded that women are dissatisfied with their body image, especially in the third trimester, when all parts of the body are affected. Dissatisfaction with body image during pregnancy was habitually accompanied by adverse maternal and child health outcomes. For instance, Silveira et al. [18] and Bergmeier et al. [12] revealed that body image dissatisfaction was connected to depressive symptoms, unfavourable dieting behaviour and eating disorders, excessive gestational weight gain, and postpartum weight retention.

Rashid and Mohd [19] posited that social support acts as a protective factor against body image disturbance and pregnancy-related disorders, which may enhance subjective well-being. Furthermore, social support from friends is negatively correlated with depression, anxiety, and stress [8]. The likelihood of depression, anxiety, and self-harm during pregnancy, according to Badeso et al. [20], is significantly correlated with low social support. The authors further stated that policymakers and individuals involved in maternity care should think about creating specialized social support programs for preventing mental health problems among pregnant women. Akiki et al. [21] posited that due to the lack of a support system, pregnant women with low social support may experience stress and may later develop depression because they lack a confidant, a source of crucial information or guidance, or someone to assist them in coping with bad emotions. Social support, according to Renbarger et al. [22], comprises four functional constructs: emotional, instrumental, informational, and appraisal. These four social support concepts, according to Biaggi et al. [23], may have a buffering effect in which resources made available to a person may improve their health and lessen the negative effects of stresses to which they are exposed. Additionally, pregnant women with limited social support are less content with their families and less adept at dealing with others; as a result, they may experience loneliness, have difficulty with stress, and subsequently develop anxiety. Social support and body image perception, together with other factors, have been generally discovered in previous studies [24, 25] to be predictors of psychological distress among pregnant women, but to the best of our knowledge, no study has specifically evaluated their relationship in the third trimester in Nigeria. This study therefore determined the relationship among body image perception, social support, and psychological distress. It further predicts the moderating effects of tangible, belonging and appraisal support on the relationship between body image perception the level of psychological distress. The findings of this study may help healthcare professionals provide an intervention for pregnant women who are experiencing psychological distress during their third trimester whenever they attend the antenatal clinic.

## Method

### Research design

This study utilized a cross-sectional descriptive design.

### Study population and size

The study was conducted among third-trimester pregnant women attending two selected primary health centres and two selected teaching hospitals in the Ogbomoso area of Oyo State, Nigeria. The primary health centres are the Ibrahim Taiwo and Adebayo Alata primary health care centres, while the teaching hospitals are Bowen University Teaching Hospital and Ladoke Akintola University Teaching Hospital. The inclusive criteria are all consenting pregnant women at 29 to 40 weeks of gestation attending the hospitals between 1st March and 15th June 2022. Also, the pregnant women should not have history of mental illness or medical problem history. The number of questionnaires distributed was 269, and 246 (91%) questionnaires were fit for the analysis.

### Data collection

Researchers recruited eight research assistants for the distribution of the questionnaires. The ethical clearance was obtained from the Bowen University Teaching Hospital Ethical Committee. Two research assistants were recruited for each of the facilities. The research assistants were taught to obtain consent from the respondents and distribute the questionnaires. The Chief Medical Director of the Teaching Hospitals and the Medical Officer in Charge of the Health Centres gave their approval to collect the data from each of the facilities. The research assistant went to the hospital during the antenatal clinic days and requested that the pregnant women be within the gestational age range of interest. Convenient sampling technique was used and any consenting pregnant women met during the antenatal visit were recruited. The pregnant women were informed about the study and were told about their confidentiality and willingness to participate in it. Those who agreed to participate in the study were thereafter given the consent form and questionnaire to complete, which were collected immediately.

### Research instruments

The research instrument adopted for the purpose of this study was divided into four sections:

### Sociodemographic characteristics

This questionnaire includes questions regarding the demographic, social, and medical characteristics of women, such as age, monthly income, religion, marital status, level of education, occupation, ethnicity, and family type.

### Body image perception

The Body Image Perception Scale was adopted to measure body image perception among pregnant women [26]. It is a 49-item scale with seven (7) subscales, including body image ideals, body image importance, body dissatisfaction, body change (global and specific parts), appearance-related behaviours (appearance and physical activity), sexual attractiveness, and functioning of the body. For each of the Body Image Importance, Body Image Ideals, Sexual Attractiveness, and Functioning of the Body subscales, participants ranked how the item applied to their experience on a five-point Likert scale (1 = strongly agree through to 5 = strongly disagree). For both the Body Dissatisfaction and Body Change subscales, participants ranked their level of satisfaction with body parts or body changes on a five-point Likert scale, ranging from one (strongly satisfied) through five (strongly dissatisfied). For the appearance-related behaviours subscale, items were ranked according to the participant’s level of engagement with the behaviour, with response options ranging from ‘never engaged with the behaviour’ to ‘always engaged in the behaviour’. Item scores were summed together to produce a total score for each of the subscales; higher scores were indicative of greater disturbance of the aspect of body image (e.g., a higher score on the Body Change subscale was indicative of higher body dissatisfaction with the body changes experienced).

### Psychological distress

The Kessler Psychological Distress Scale (K10) was adopted to assess the level of psychological distress among pregnant women. It is a 10-item question rated on a five-point Likert scale ranging from 1 (none of the time) to 5 (all of the time). It has a minimum score of 10 and a maximum score of 50. Low scores indicate low levels of psychological distress, and high scores indicate high levels of psychological distress. It has been documented that a score of 0–24 indicates less psychological distress, while a score of 25–50 indicates significant psychological distress [27]. The continuous score was used for the analysis. The Cronbach alpha of the instrument from a previous study is 0.88.

### Social support

A shortened version of the Interpersonal Support Evaluation Scale consisting of 12-item questions was used to measure the level of perceived social support received by the pregnant women [28]. The question is measured on a 4-point Likert scale ranging from “definitely true” to “definitely false.” This questionnaire has three different subscales designed to measure three dimensions of perceived social support, including appraisal support, belonging support, and tangible support. Items 2, 4, 6, and 11 make up the Appraisal Support Subscale; Items 1, 5, 7, and 9 make up the Belonging Support Subscale; and Items 3, 8, 10, and 12 make up the Tangible Support Subscale. The Cronbach values for appraisal support, belonging support, and tangible support in this study are 0.86, 0.82, and 0.85, respectively.

### Method of data analysis

Statistical Package for Social Sciences (version 23) was used to analyze the data. Both descriptive statistics (frequency and percentages) and inferential statistics (ANOVA and multiple regression) were carried out. The social demographic variables were described by frequency and percentages. The relationship between the sociodemographic variables and psychological distress among the pregnant women were determined by ANOVA. Multiple regression including moderation analysis using robust standard errors was conducted using psychological distress as dependent variable while body image perception, tangible support, belonging support and appraisal support as independent variable. The demographic variables including, religion, marital status, level of occupation, ethnicity and family type were included in regression as the control variables. For the moderating effect of tangible support, belong support and appraisal support on the relationship between body image perception and psychological distress,, three interaction terms were created which are (1)tangible support*body image perception; to determine the effect of tangible support on the relationship between the body image perception and psychological distress (2) belonging support*body image perception; to determine the effect of belonging support on the relationship between body image perception and psychological distress and (3) Appraisal support*body image perception ; to determine the effect of appraisal support on the relationship between body image perception and psychological distress.

## Result

Table 1 shows the demographic variables of the pregnant women in the third trimester. The mean age and standard deviation were 26 years and 5 years, respectively. One hundred and fifty (61%) pregnant women were Christians, and thirty-one (12.6%) were single. Only a few (4.1%) had no formal education, and one hundred forty-two (57.7%) had secondary education. The majority (70.3%) were self-employed, and 70 (28.5%) and one hundred and ninety-three (78.5%) were from the Yoruba ethnic group. Most of the pregnant women (72.4%) were from nuclear families, while the average monthly income of the pregnant women was $60,000. The relationship between sociodemographic variable of the pregnant women and the psychological distress in Table 2 reveals the significant relationship between family type, occupation, level of education and psychological distress In Table 3, Being married was associated with lower psychological distress by 0.2 average point. Any problem with body image perception was associated with psychological distress by 1.6 score. Utilizing appraisal support reduced the level of psychological distress by 1.9 while belonging support reduces the psychological distress by 2.1. Tangible support also reduced the psychological distress by average of 1.0. The interaction terms (Appraisal support*body image perception, belonging support*body image perception and belonging support*body image perception) reveals that the coefficient was negative which means that the effect of belonging, appraisal and tangible support on body image perception was negative and were statistically significant. This implies that the presence of various forms of social support reduces the positive relationship between body image perception and psychological distress of the pregnant women. In the presence of tangible support, the coefficient on body image perception is reduced by 0.2. This implies that pregnant women with psychological distress due to body image perception have a lower score of 1.37, while in the presence of belonging support had a lower score of 0.34. Psychological distress in pregnant women due to body image perception had a lower score of 0.79 points in the presence of appraisal support.

**Table 1:** Sociodemographic variables of pregnant women during the third trimester

VARAIBLE		*N*(246)	%
AGE	39.34 ± 11.28		
MONTHLY INCOME	60.098 ± 9.344		
RELIGION	CHRISTIAN	150	61.0
	MUSLIM	96	39.0
MARITAL STATUS	SINGLE	31	12.6
	MARRIED	215	87.4
LEVEL OF EDUCATION	NO FORMAL EDUCATION	10	4.1
	PRIMARY	5	2.0
	SECONDARY	142	57.7
	TERTIARY	89	36.2
OCCUPATION	SELF-EMPLOYED	173	70.3
	EMPLOYED	70	28.5
	RETIRED	3	1.2
ETHNICITY	HAUSA	11	4.5
	YORUBA	193	78.5
	IGBO	30	12.2
	OTHERS	12	4.9
FAMILY TYPE	NUCLEAR FAMILY	178	72.4
	EXTENDED FAMILY	68	27.6

**Table 2:** Differences in psychological distress to sociodemographic variables

		*n*/%	t/f test (p)	
Variables				
Age	15–24	46(18.7)	2.34(0.782)	
	25–34	151(61.4)		
	35–44	39(15.9)		
	≥ 45	10(4.0)		
Monthly income	< 50,000	21(8.5)	1.45(0.663)	
	51,000-100,000	107(43.5)		
	101,000-150,000	95(38.6)		
	≥ 151,000	23(9.3)		
Religion	Christian	150 (61)	**4.22(0.003)**	
	Muslim	96(39)		
Marital Status	Single	31(12.6)	**5.39(0.043)**	
	Married	215(87.4)		
Level of Education	No formal education	10(4marital status, and religion.1)		
	Primary	5(2.0)		
	Secondary	142(57.7)	**3.89(0.026)**	
	Tertiary	89(36.2)		
Occupation	Self-employed	173(70.3)	**8.35(0.002)**	
	Employed	70(28.5)		
	Retired	3(1.2)		
Family type	Nuclear family	178(72.4)	**11.78(0.04)**	
	Extended family	68(27.6)		
Ethnicity	Hausa	11(4.5)		
	Yoruba	193(78.5)	5.88(0.85)	
	Igbo	30(12.2)		
	Others	12(4.9)		

**Table 3:** Regression of body image perception, tangible support, belonging support, appraisal support and control variables on psychological distress of pregnant women

Variables	B	Robust SE	β	t	*P*
Religion	-0.03	0.05	-0.03	-0.65	0.824
Marital Status	-0.21	0.07	-0.19	-3.26	< 0.001
Level of Education	-0.05	0.06	-0.04	-2.13	0.228
Occupation	-0.04	0.03	-0.02	-1.28	0.137
Ethnicity	0.00	0.06	0.01	0.06	0.986
Family type	0.22	0.10	0.08	2.09	0.065
Body Image Perception	1.57	0.03	-0.25	-4.66	**< 0.001**
Appraisal Support	-1.85	0.06	0.12	-3.23	**< 0.001**
Belonging support	-2.08	0.04	0.62	4.24	**< 0.001**
Tangible support	-1.03	0.01	0.40	8.23	**< 0.001**
Appraisal Support*Body Image perception	-0.78	0.39	-0.29	0.17	**< 0.001**
Belonging support*Body image perception	-1.23	0.02	-0.42	0.20	**< 0.001**
Tangible support*Body Image perception	-0.17	0.01	-0.37	0.11	**< 0.001**

R^2^ = 0.44, Adjusted R^2^ = 0.38, F(13, 238) = 12.06, *p* < 0.001

Dummy variables: Religion (Christian 1; Muslim 0), Marital status (single 1, married 0), Level of education (No formal education, primary, secondary; 1 Tertiary 0) Occupation (self employed 1, employed and Retired 0); Ethnicity (Yoruba, 1, Hausa, Igbo 0), Family type (Nuclear family 1, Extended family 0)

## Discussion of findings

This study was conducted to explore the relationship between body image perception, social support, and psychological distress among pregnant women in the third trimester. Findings from this study show that the greater the extent to which pregnant women are dissatisfied with their body image perception, the more they experience psychological distress. This is in line with Linde and colleagues, who reported women’s dissatisfaction with different components of body image perception during the second and third trimesters [29]. It also corroborated the findings of Yasemei and colleagues that pregnant women’s psychological distress levels decreased when they felt themselves as powerful in a sexual relationship [30].

Findings from this study further revealed that pregnant women who are dissatisfied with body change and appearance-related behaviour, experienced a high level of psychological distress. This implies that the more a woman is satisfied with her body change and appearance during pregnancy, the more she experiences psychological stability and wellbeing. It also means that a negative perception of body changes and appearance-related behaviour during pregnancy can result in psychological distress, which can inadvertently influence the progress and outcome of pregnancy. Przybyla-Basista et al. [31] further confirmed this in a study of 150 Polish pregnant women higher levels of negative evaluation of appearance increased the chances of depression in pregnant women by almost one and a half. Linde [29] reported that body dissatisfaction during pregnancy can affect maternal and foetal health, prompting women to severely restrict their eating or triggering a relapse of an eating disorder, and the risk of perinatal depression is four times higher in women who are dissatisfied with their body image. Ojha & Kumar [32] further revealed that a positive body image can improve self-esteem, satisfaction, and psychological wellbeing, while body image dissatisfaction can result in unhappiness.

According to Kowalska [33], social support is significantly related to the level of perceived stress and anxiety experienced by pregnant women. Findings from this study revealed that all three components of social support reduce the level of psychological distress though at different level. Evidence has shown that appraisal of life events improves mental health [34], using it for pregnant women who are dissatisfied with body change will produce appropriate results. Consistent with a previous study, our findings further revealed that tangible and belonging social support are essential for improving the psychological health of pregnant women during the late stage of pregnancy. According to our findings, singles experienced less psychological distress than married individuals. Marital status is a major sociodemographic component influencing both the physical and psychological health of pregnant women. Pregnant women with higher social support from spouses are healthy mentally [35], while poor relationships with husbands are a predisposing factor to prenatal depression [36].

The clinical implication of this study is that the psychological distress experienced by pregnant women can be reduced with the appropriate use of social support components, and this should be emphasized during the antenatal clinic. Healthcare providers should ask pregnant women about their feelings about their body changes and the various components of social support they receive. Also, specialists in psychotherapy and counselling should be engaged during the antenatal clinic for pregnant women who are prone to poor body image perception.

The limitation of this study is the use of a quantitative study design only. The mixed method, including both quantitative and qualitative design, will provide in-depth insight into the relationship among the variables. The strength of this study is the specific group of pregnant women considered who are in the third trimester and have obvious changes in body shape and size.

## Conclusions

Pregnancy and childbirth bring a variety of changes to a woman, affecting every aspect of her life. Some women embrace the changes that come with pregnancy and giving birth, but for many, metamorphosis can negatively shift their self-image. This study revealed that pregnant women who are concerned about their body image perception has increase level of psychological. Appraisal, tangible and belonging social support reduced the level of psychological distress among the pregnant women. It is therefore recommended that intervention programs focusing on bolstering appraisal, tangible and belonging support be provided for pregnant women in the third trimester.

## Data Availability

The datasets used and/or analysed during the current study are available from the corresponding author on reasonable request.
